# About the Other Side of Sleep Disorders: Improving Daytime Problems, Fatigue, Sleepiness, Cognitive Functioning and More

**DOI:** 10.1111/jsr.70164

**Published:** 2025-08-02

**Authors:** Susanna Jernelöv, Kerstin Blom

**Affiliations:** ^1^ Centre for Psychiatry Research, Department of Clinical Neuroscience Karolinska Institutet, & Stockholm Health Care Services Stockholm Sweden; ^2^ Division of Psychology, Department of Clinical Neuroscience Karolinska Institutet Stockholm Sweden

**Keywords:** cognitive behavioural therapy for insomnia (CBT‐i), daytime functioning, insomnia

## Abstract

Daytime impairments—such as fatigue, emotional instability, and cognitive difficulties—are increasingly acknowledged as core features of insomnia, yet they remain underrepresented in both research and treatment strategies. While CBT‐I remains the gold standard for treating nocturnal symptoms, its effects on daytime functioning, which are often the primary concern for patients, are less robust, inconsistently measured, and poorly understood. This narrative review highlights the need to elevate daytime symptoms from secondary outcomes to central targets in both research and clinical practice. Key gaps include the lack of standardised and conceptually clear outcome measures, limited personalisation of CBT‐I protocols, and insufficient understanding of the mechanisms linking improved sleep to daytime recovery. Moreover, the ethical implications of emerging digital assessment tools must be addressed to ensure that technological innovation does not come at the cost of participant trust or autonomy. To move the field forward, future research should prioritise daytime functioning as a primary endpoint, adopt ethically grounded multimodal assessment strategies, and explore adaptive, symptom‐specific treatment designs. Finally, insomnia should be recognised not only as a disorder in its own right but also as a transdiagnostic and potentially preventable contributor to broader mental health problems. Addressing these challenges may lead to more effective, personalised, and patient‐centred care for individuals living with insomnia.

## Graphical abstract

This review highlights insomnia's underexplored daytime consequences. While CBT‐I effectively treats nocturnal symptoms, its impact on daytime functioning remains limited. To move the field forward, we argue for elevating daytime symptoms as primary targets and propose personalised, ethical, and symptom‐specific strategies to advance treatment and align with patient needs.

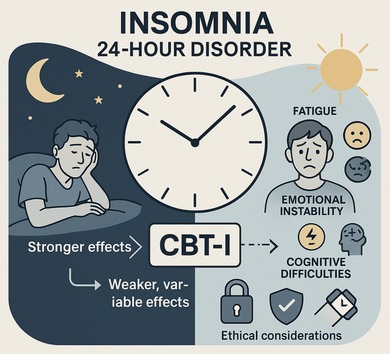

## 1 Introduction

Insomnia is one of the most prevalent sleep disorders, affecting approximately 10% of the population in its chronic form (Morin et al. 2015; van Straten et al. 2025). Increasingly, insomnia is conceptualised as a 24‐h disorder, where daytime symptoms—such as fatigue, impaired concentration, mood disturbances, and reduced quality of life—are not only common but essential for diagnosis according to DSM‐5 criteria (American Psychiatric Association 2013).

Despite this, research has historically focused on nocturnal symptoms and objective sleep parameters, while daytime impairments have often been treated as secondary outcomes. This imbalance is reflected in the literature, where most randomised controlled trials of insomnia interventions prioritise sleep onset latency, wake after sleep onset, and sleep efficiency, rather than functional outcomes during waking hours.

Cognitive Behavioural Therapy for Insomnia (CBT‐I) is the recommended first‐line treatment for insomnia in both American (Edinger et al. 2021) and European clinical guidelines (Riemann et al. 2023). CBT‐I has demonstrated robust effects on nocturnal symptoms and sleep‐related cognitions, but its impact on daytime functioning remains less clear. A comprehensive meta‐analysis by Benz et al. (2020) identified 10 categories of daytime symptoms and found that CBT‐I yields small to moderate improvements across domains such as fatigue, sleepiness, depression, anxiety, and social functioning—effects that are notably weaker than those observed for nocturnal symptoms.

Meanwhile, pharmacological treatments for insomnia, including agents such as zolpidem, eszopiclone, and suvorexant, have shown efficacy in improving sleep parameters but are associated with adverse effects that may impair daytime functioning. These include residual sedation, cognitive slowing, and increased fall risk, particularly in older adults (Wickwire et al. 2016). This underscores the importance of evaluating how non‐pharmacological interventions like CBT‐I affect daytime symptoms, not only as a measure of treatment efficacy but also as a potential safeguard against iatrogenic harm.

In this review, we summarise current knowledge on the effects of CBT‐I on daytime symptoms, highlight gaps in the literature, and propose directions for future research. We argue that daytime symptoms should be elevated from secondary outcomes to central targets in both clinical trials and treatment design, particularly as they often represent the primary reason patients seek help.

## 2 What Do we Know Today?

### 2.1 A Growing Evidence Base for CBT‐I and Daytime Symptoms

Insomnia has long been defined as a disorder involving both nocturnal and daytime symptoms (American Psychiatric Association 2013), and when developing the initial components of CBT‐I, sleep restriction therapy, Arthur Spielman and colleagues (Spielman, Caruso, and Glovinsky 1987; Spielman, Saskin, and Thorpy 1987) proposed that daytime functioning could serve as a clinically meaningful indicator for determining optimal sleep window duration during sleep restriction therapy—as opposed to primarily using sleep efficiency as an indicator. However, for a long time, research has prioritised nighttime complaints.

In recent years, attention has gradually shifted back to the clinical relevance of daytime impairments, which is particularly important as they often represent the primary reason patients seek treatment (Morin et al. 2006).

A pivotal contribution to this shift was made by Benz et al. (2020), who conducted a systematic review and network meta‐analysis of 86 randomised controlled trials (RCTs) involving over 15,000 participants. Their analysis identified 10 distinct categories of daytime symptoms commonly assessed in insomnia research: depression, anxiety/worry, daytime sleepiness, fatigue, quality of life, daytime and social functioning, physical functioning, mental state, stress, and cognitive functioning.

Across these domains, CBT‐I demonstrated statistically significant effects, with small to moderate effect sizes (Cohen's d = 0.2–0.5). The strongest effects were observed for depressive symptoms, fatigue, and daytime functioning, particularly when CBT‐I was delivered individually face‐to‐face or via supported internet‐based formats. However, these effects were consistently smaller than those reported for nocturnal symptoms, suggesting that improvements in daytime functioning may be secondary to improved sleep rather than a direct result of targeted intervention.

### 2.2 Depression and Anxiety: Dual Roles as Symptoms and Diagnoses

Among the most frequently studied daytime symptoms in insomnia research are depression and anxiety. These conditions are often seen both as daytime manifestations of insomnia, such as low mood, irritability, or excessive worry, and as full psychiatric diagnoses with 24‐h impact. This duality complicates interpretation of treatment outcomes, particularly in studies evaluating the effects of Cognitive Behavioural Therapy for Insomnia (CBT‐I).

For instance, improvements in depressive symptoms following CBT‐I may reflect reduced sleep‐related distress, enhanced emotion regulation due to better sleep, or indirect effects on comorbid depression. Palagini et al. (2024) provide compelling evidence that CBT‐I may serve not only as a treatment for insomnia but also as a preventive intervention for mood disorders. Their meta‐analysis found that CBT‐I significantly reduced subclinical depressive symptoms and may delay or prevent the onset of major depressive episodes, particularly in high‐risk populations.

However, the therapeutic boundaries between insomnia and depression remain blurred. Blom et al. (2024) conducted a double‐blind randomised controlled trial comparing a combined CBT‐I and CBT for depression protocol to CBT for depression with a placebo insomnia intervention. While CBT‐I showed specific and robust effects on insomnia severity, the combined treatment did not yield superior outcomes for depression compared to CBT for depression alone. This suggests that the antidepressant effects of CBT‐I may be general rather than additive or synergistic when combined with targeted depression treatment.

These findings raise important questions about the mechanisms through which CBT‐I influences mood. Palagini et al. (2024) propose that insomnia contributes to mood dysregulation via hyperarousal, HPA‐axis activation, and inflammatory processes—mechanisms that CBT‐I may help normalise. This biological perspective supports the idea that insomnia is not merely a symptom of depression but a modifiable risk factor with transdiagnostic relevance.

Moreover, longitudinal studies have shown that insomnia often precedes the onset of depression and anxiety, reinforcing its role as a prodromal condition (Hertenstein et al. 2019; Johansson et al. 2021). This temporal relationship underscores the need to treat insomnia not only to improve sleep but also to mitigate broader mental health risks. Indeed, it has been shown that treating insomnia with CBT‐I may be able to prevent the onset of depression (Boland et al. 2023; Cheng et al. 2019; Irwin et al. 2021).

Important but mostly overlooked is that not only affective disorders and anxiety, but also other psychiatric conditions such as ADHD and psychotic disorder may serve similar dual roles, as sleep status may affect core symptoms such as attention (Wüst et al. 2024) and hallucinations (Sheaves et al. 2016), and paranoia may improve with CBT‐i (Freeman et al. 2017).

## 3 Measurement Challenges and Gaps

Despite growing interest in daytime outcomes, standardised measurement remains lacking. Most studies rely on self‐report instruments, which vary in quality and are prone to bias. Objective assessments—such as neuropsychological testing—are rare, and results are often inconsistent due to methodological heterogeneity (Wardle‐Pinkston et al. 2019).

Recent advances in wearable technology and digital phenotyping offer new possibilities for capturing behavioural rhythms, mobility, and alertness. These tools can passively collect large volumes of metadata, including movement, geolocation, and sleep–wake patterns, often without active user engagement. While promising, they raise ethical concerns around privacy, consent, and algorithmic bias (D'Alfonso et al. 2025).

To safeguard participant autonomy and trust, future research must establish robust ethical frameworks. This includes transparent consent procedures, data minimisation, and participant control over data use and deletion. Researchers must also reflect on key ethical questions: Would participants still consent if they understood the full scope of data collection? Are we collecting only what is necessary? Can participants grasp how their data flows through the system? Are some groups more vulnerable to harm? What unintended consequences might arise?

Without such safeguards, the promise of objective measurement may come at too high a cost—particularly in vulnerable populations with psychiatric comorbidities. As digital tools become more prevalent, ethical standards must evolve in parallel.

## 4 Recent Research and Expanding Focus

Since the publication of Benz et al. (2020), several new studies and meta‐analyses have expanded our understanding of how CBT‐I affects daytime functioning. These contributions have helped to clarify both the scope and limitations of current evidence, while also pointing towards new directions for research.

A few years ago, Alimoradi et al. (2022) conducted a systematic review and meta‐analysis focusing on the effects of CBT‐I on quality of life. They found that CBT‐I significantly improves quality of life across multiple domains, with moderate effect sizes. These findings reinforce the notion that the benefits of CBT‐I extend beyond sleep itself and into broader aspects of daily functioning, although—again—the mechanisms remain unclear.

A recent component network meta‐analysis by Furukawa, Sakata, Yamamoto, et al. (2024) examined which elements of CBT‐I contribute most to treatment outcomes. Their findings suggest that sleep restriction therapy (SRT) and stimulus control are the most effective components for improving both nocturnal and daytime symptoms.

Daytime sleepiness, a particularly salient symptom for many patients, has also been the focus of some experimental work. Maurer et al. (2020) investigated the acute effects of sleep restriction therapy on circadian timing and vigilance. Their results indicate that SRT can lead to short‐term impairments in vigilance, particularly in the early stages of treatment. This highlights a potential trade‐off: while SRT may consolidate sleep and improve long‐term outcomes, it may also temporarily exacerbate certain daytime symptoms. These findings underscore the importance of monitoring daytime functioning throughout treatment—not just as an endpoint, but as a dynamic variable that may fluctuate in response to therapeutic interventions.

Perhaps most notably, Morin et al. (2023) performed a large randomised clinical trial comparing psychological and pharmacological treatments for insomnia to investigate effects on daytime functions. The study found that CBT‐I and medication both improved sleep outcomes, but only CBT‐I led to sustained improvements in daytime functioning at follow‐up. Moreover, pharmacological treatments were associated with more frequent reports of residual daytime sedation and blunted cognitive responsiveness—side effects that may undermine functional recovery despite improved sleep metrics.

Together, these studies suggest that CBT‐I has a measurable, though variable, impact on daytime symptoms such as fatigue, sleepiness, mood, and quality of life. Please see Table 1 for an illustrative summary of the findings.

**TABLE 1:** Illustrative summary of frequency and effects of CBT‐I on different daytime symptoms.

Daytime symptom category	Commonly studied	Reported effect size (CBT‐I)	Comments
Anxiety/worry	Yes	Small to moderate	High heterogeneity, interpretation complicated by comorbidity with depression; improvements may reflect reduced sleep‐related worry or broader emotional regulation
Cognitive functioning	No	—	Measurement challenges and heterogeneity
Depression	Yes	Small to moderate	Interpretation complicated by overlap with clinical depression; may reflect both symptom relief and broader mood effects.
Daytime sleepiness	Not very common	Small	May worsen temporarily during SRT
Daytime/social functioning	Not very common	Small to moderate	Often self‐reported; fewer studies using objective data
Fatigue	Quite common	Small to moderate	Consistent improvements across studies, but may worsen during SRT
Mental state	Not very common	Small	Conceptually diffuse category
Physical functioning	Quite rare	Not statistically significant	No effects found, less commonly assessed
Quality of life	Quite rare	Small to moderate	Improvements across multiple domains, but low power and heterogeniety
Stress	Rare	Not statistically significant	No effects found, the concept of “stress” often overlaps with anxiety

*Note*: Illustrative summary of daytime symptom categories identified in Benz et al. (2020) and their reported responsiveness to CBT‐I. This table is based on a narrative synthesis of findings presented in the current manuscript and is not derived from a systematic review. Effect sizes are approximate and intended to provide a general overview rather than precise estimates.

## 5 What is Missing?

Despite the growing body of research on CBT‐I and daytime symptoms, several critical gaps remain. These limitations span conceptual, methodological, and clinical domains, and addressing them will be essential for advancing both science and practice.

1. Daytime Symptoms as Primary Outcomes

One of the most persistent limitations in insomnia research is the continued treatment of daytime symptoms as secondary or exploratory outcomes in clinical trials. Despite the fact that diagnostic criteria for insomnia disorder explicitly include daytime impairments such as fatigue, cognitive difficulties, and mood disturbances, most studies still prioritise nocturnal metrics like sleep onset latency, wake after sleep onset, and total sleep time. This reflects a longstanding sleep‐centric paradigm, where the implicit assumption is that improving sleep at night will naturally lead to better functioning during the day.

While this assumption may hold in some cases, emerging evidence suggests that the relationship between nocturnal improvements and daytime recovery is neither linear nor guaranteed. While interventions like CBT‐I produce robust and long‐term effects on subjective sleep complaints and sleep parameters (Furukawa, Sakata, Furukawa, et al. 2024), their impact on daytime functioning tends to be smaller and more variable (Benz et al. 2020). This discrepancy raises important questions about the mechanisms of change and whether current interventions adequately address the full spectrum of insomnia‐related impairment.

Moreover, by relegating daytime symptoms to secondary status, studies may be underpowered to detect meaningful changes in these domains or may fail to capture the outcomes that matter most to patients. Many individuals seek treatment not because they are awake at night, but because they are exhausted, unfocused, or emotionally dysregulated during the day. Elevating daytime functioning to a primary outcome would not only align research with patient priorities but also encourage the development of more comprehensive and targeted interventions.

2 Conceptual Clarity

A major challenge in insomnia research is the lack of consensus on what constitutes ‘daytime functioning’. Terms such as fatigue, sleepiness, cognitive impairment, and quality of life are frequently used interchangeably, despite referring to distinct constructs with different physiological underpinnings and clinical implications. This conceptual ambiguity complicates both the measurement of outcomes and the interpretation of treatment effects. For instance, fatigue and sleepiness are often conflated, yet they reflect different mechanisms where fatigue is more related to perceived effort and energy depletion while sleepiness reflects a physiological drive to sleep. They may thus respond differently to interventions like CBT‐I or pharmacotherapy.

This lack of definitional precision also affects the selection of outcome measures. Studies may use broad or overlapping instruments, making it difficult to compare findings across trials or to isolate which aspects of daytime functioning are most responsive to treatment. Without clear conceptual boundaries, it becomes challenging to determine whether observed improvements are due to direct effects on specific symptoms or indirect effects mediated by improved sleep.

Moreover, as mentioned above, some symptoms—such as depression and anxiety—blur the line between daytime complaints and full psychiatric disorders. While they are often included in insomnia studies as secondary outcomes, they also represent independent diagnostic entities with 24‐h impact. This dual role raises important questions about causality, comorbidity, and treatment specificity. For example, does CBT‐I reduce depressive symptoms by improving sleep, or does it also target underlying mood dysregulation? And are improvements in anxiety a byproduct of reduced sleep‐related worry, or does it represent a broader therapeutic effect (Baglioni et al. 2011; Hertenstein et al. 2019)?

Clarifying these conceptual distinctions is essential for advancing both research and clinical practice. It would allow for more precise targeting of interventions, better alignment between patient‐reported concerns and study outcomes, and a clearer understanding of how insomnia interacts with broader domains of mental and physical health.

3 Measurement Improvement

As discussed earlier, the field lacks standardised, reliable, and validated tools for assessing daytime symptoms in insomnia populations. Most studies rely on self‐report instruments, which are not without merit. However, given the significance of patients' subjective experiences, it is particularly concerning that several existing questionnaires, such as the Sleep Need Questionnaire, lack thorough psychometric evaluation. Moreover, subjective measures may fail to detect subtle or domain‐specific changes. Consequently, objective methods such as neurocognitive testing or actigraphy remain underutilised and are often applied inconsistently.

Furthermore, while wearable technologies and digital phenotyping offer exciting possibilities for continuous, real‐world assessment of daytime functioning, they also raise significant ethical concerns. Passive data collection through GPS tracking, accelerometry, or smartphone usage patterns may infringe on personal privacy and autonomy, particularly if implemented without robust consent procedures and data protections. These methods may be acceptable in tightly controlled research settings, but their use in clinical practice must be guided by clear ethical frameworks to avoid undermining trust and patient agency.

4 Personalisation and Mechanistic Insight

Despite the growing sophistication of insomnia research, many CBT‐I protocols remain relatively standardised. On the one hand, this makes them relatively scalable, as they may be easily taught to health‐care staff with limited training in CBT or sleep. However, this also means they offer limited flexibility to tailor treatment based on individual symptom profiles. This “one‐size‐fits‐all” approach may overlook important variations in how different patients experience and respond to insomnia and insomnia treatment. For example, some individuals may present primarily with cognitive hyperarousal and benefit most from cognitive restructuring, while others may struggle with irregular sleep schedules and respond better to behavioural components like sleep restriction or stimulus control.

Yet, few studies have systematically examined whether specific components of CBT‐I are more effective for particular daytime symptoms, such as fatigue, concentration difficulties, or emotional dysregulation (Furukawa, Sakata, Yamamoto, et al. 2024). This gap in the literature limits our ability to tailor interventions to individual symptom profiles, which may partly explain the modest and variable effects observed on daytime functioning.

Equally underexplored are the mechanisms through which CBT‐I exerts its effects on daytime outcomes. Is improvement in fatigue mediated by increased sleep efficiency? Does reduced pre‐sleep worry enhance cognitive performance during the day? Could changes in circadian alignment influence mood regulation? These are empirical questions that remain largely unanswered, in part due to the limited use of mediation analyses and mechanistic study designs in the field.

Understanding these pathways is not only of theoretical interest—it has practical implications for optimising treatment. A more mechanistically informed and symptom‐targeted approach could enhance both efficacy and efficiency, particularly in complex or comorbid cases. Future research should prioritise dismantling studies, component analyses, and adaptive designs that allow for personalisation based on baseline symptom profiles and treatment response trajectories.

## 6 Future Directions: Toward a Daytime‐Centred Model of Insomnia Research

The growing recognition of insomnia as a 24‐h disorder calls for a corresponding shift in research priorities. To fully understand and treat the disorder, future studies must place greater emphasis on daytime symptoms—not only as secondary outcomes, but as central indicators of treatment success, patient well‐being, and functional recovery.

1. Elevating Daytime Symptoms to Primary Outcomes

To better reflect the lived experience of individuals with insomnia, future clinical trials could be designed with daytime functioning as a primary endpoint rather than a secondary consideration, or perhaps with sleep and daytime function as dual primary outcomes. This includes pre‐registering daytime outcomes, ensuring adequate statistical power to detect changes in these domains, and selecting validated, sensitive instruments that can capture subtle but meaningful improvements in areas such as fatigue, cognitive performance, mood, and social functioning.

Future research should distinguish between patients with subclinical symptoms and those with diagnosed psychiatric disorders. It is possible that CBT‐I is more effective as a preventive or early intervention than as a treatment for established psychiatric conditions, but support for such a statement is still scant. Clarifying the conceptual and diagnostic boundaries between insomnia‐related symptoms and comorbid disorders will be essential for optimising treatment strategies and improving patient outcomes.

Prioritising daytime outcomes would align research more closely with patient‐centred goals. Many individuals do not seek treatment solely because they struggle to fall or stay asleep, but because these nocturnal difficulties impair their ability to function during the day, leaving them exhausted, unfocused, emotionally dysregulated, or socially withdrawn. By elevating daytime symptoms to primary status, researchers can better evaluate the real‐world impact of interventions and develop treatments that target what matters most to patients.

Moreover, treating daytime functioning as a core outcome could help shift the field toward a more holistic, 24‐h model of insomnia, where both night and day are considered integral to not only diagnosis, but also treatment and recovery. This shift would also encourage the development of novel interventions or adaptations of CBT‐I that more directly address daytime impairments, potentially improving both efficacy and patient satisfaction.

2 Developing Ethical, Multimodal Assessment Strategies

To advance the measurement of daytime functioning in insomnia research, future studies should adopt multimodal assessment strategies that integrate self‐report instruments, behavioural tasks, and passive sensing technologies. This layered approach can provide a more comprehensive and ecologically valid picture of how insomnia affects individuals across different domains of daily life.

However, the use of such technologies must be grounded in strong ethical principles. Passive data collection through wearables, smartphones, or digital phenotyping tools can yield rich insights into activity patterns, sleep–wake rhythms, and cognitive or emotional states, but it also introduces significant risks related to privacy, autonomy, and data security. Transparent and meaningful informed consent is essential, ensuring that participants understand what data is being collected, how it will be used, and who will have access to it.

In addition, researchers should apply data minimisation principles, that is, collecting only what is necessary for the research question, and provide participants with control over their data, including options to withdraw or delete it. These safeguards are particularly important when working with vulnerable populations, such as individuals with psychiatric comorbidities, who may be even more susceptible to harm from data misuse or breaches of confidentiality.

Ultimately, the promise of digital tools must be balanced with a commitment to ethical integrity. Multimodal assessment strategies should not only enhance scientific precision but also uphold the values of respect, transparency, and proportionality in research design and implementation.

3 Personalising Treatment Based on Daytime Profiles

There is a growing recognition that “one‐size‐fits‐all” CBT‐I protocols may not fully address the diverse symptom profiles of individuals with insomnia. While standard CBT‐I is effective for many, its uniform structure may overlook opportunities to enhance outcomes. One way forward that may align well with needs for scalable treatments (Baglioni et al. 2023) is to adapt treatments to the generic difficulties of specific patient groups. This concept has been piloted in several patient groups (Cassel et al. 2022; Jernelöv et al. 2022; Jernelöv et al. 2019). However, even higher personalisation can be achieved by aligning treatment components with specific individual impairments, both night‐time and daytime.

Future research should thus explore whether certain elements of CBT‐I are more effective for particular dysfunction constellations. For instance, patients experiencing prominent fatigue might benefit from integrating behavioural activation strategies into the CBT‐I framework. Conversely, individuals with high levels of worry or cognitive hyperarousal may respond better to an emphasis on cognitive restructuring or mindfulness‐based techniques.

This line of inquiry could be advanced through adaptive treatment designs (Forsell et al. 2019), stepped care models (Baglioni et al. 2023) and daytime symptom‐guided dosing or titration of interventions like sleep restriction (Jernelöv 2025), following the notion that treatment duration and structure may need to be adapted not only based on nocturnal symptoms, but also on individual trajectories of daytime recovery. This aligns with the recently proposed SONA theory (Scott and Perlis 2025), which conceptualises sleep health as a dynamic interplay between sleep opportunity, sleep ability, and individual sleep need—the latter often best inferred from daytime functioning.

These approaches allow for dynamic adjustment of treatment intensity or focus based on individual response patterns, potentially improving both efficacy and efficiency. Component network meta‐analyses, like that of Furukawa, Sakata, Furukawa, et al. (2024) and Furukawa, Sakata, Yamamoto, et al. (2024), provide a foundation for this work by identifying which CBT‐I elements contribute most to overall outcomes, but further studies are needed to link these components to specific daytime benefits.

Ultimately, a more personalised CBT‐I model could lead to more targeted, personalised care—especially for individuals with comorbid conditions or treatment‐resistant symptoms.

4 Integrating Transdiagnostic and Preventive Perspectives

Insomnia should not be viewed solely as a disorder of sleep, but as a transdiagnostic condition with implications that extend across multiple domains of mental health. Daytime symptoms such as low mood, cognitive difficulties, and emotional dysregulation are not unique to insomnia—they are also early warning signs of depression, anxiety, and burnout. These overlapping features suggest that insomnia may serve as both a marker and a modifiable risk factor for broader psychopathology.

To enhance the preventive potential of CBT‐I, future interventions should prioritise early identification and targeted treatment of insomnia‐related symptoms. By addressing sleep disturbances before they escalate, there is an opportunity to reduce the risk of developing or relapsing into mood and anxiety disorders. Integrating CBT‐I into broader mental health prevention strategies could help interrupt the trajectory toward more severe or chronic psychiatric conditions.

This perspective aligns with a growing body of research advocating for transdiagnostic approaches in mental health care, where interventions are designed not only to treat specific disorders but also to reduce shared vulnerabilities across diagnostic categories (Dalgleish et al. 2020). CBT‐I, with its focus on behavioural regulation, cognitive restructuring, and emotion‐related arousal, is well‐positioned to play a role in such models.

Future research should explore the preventive potential of CBT‐I more explicitly, particularly in high‐risk populations such as adolescents, individuals with subthreshold symptoms, or those with a family history of mental illness. Doing so could help shift the field toward a more proactive, resilience‐oriented model of care.

## 7 Conclusion

Insomnia is increasingly understood as a 24‐h disorder, yet research and treatment paradigms have not fully caught up with this perspective. While CBT‐I remains the gold standard for treating nocturnal symptoms, its effects on daytime functioning—often the primary concern for patients—are less robust, inconsistently measured, and poorly understood. This review highlights the need to elevate daytime symptoms from secondary outcomes to central targets in both research and clinical practice.

Key gaps include the lack of standardised and conceptually clear outcome measures, limited personalisation of CBT‐I protocols, and insufficient understanding of the mechanisms linking improved sleep to daytime recovery. Moreover, the ethical implications of emerging digital assessment tools must be addressed to ensure that technological innovation does not come at the cost of participant trust or autonomy.

To move the field forward, future research should prioritise daytime functioning as a primary endpoint, adopt ethically grounded multimodal assessment strategies, and explore adaptive, symptom‐specific treatment designs. Finally, insomnia should be recognised not only as the debilitating disorder it is, but also as a transdiagnostic and potentially preventable contributor to broader mental health problems. Addressing these challenges may lead to more effective, personalised, and patient‐centred care for individuals living with insomnia.

## Author Contributions

**Susanna Jernelöv:** conceptualization, writing – original draft, methodology, visualization, writing – review and editing, investigation, project administration. **Kerstin Blom:** writing – original draft, methodology, writing – review and editing, investigation, project administration.

## Ethics Statement

The authors have nothing to report.

## Conflicts of Interest

The authors declare no conflicts of interest.

## Data Availability

Data sharing is not applicable to this article as no new data were created or analyzed in this study.
